# Effect of Dual-task Standing on prefrontal-motor Cortex Activation and postural-related Muscle Activity between Young and Older Adults

**DOI:** 10.1007/s10548-025-01137-8

**Published:** 2025-09-11

**Authors:** Jiahao Pan, Shuqi Zhang

**Affiliations:** 1 School of Physical Education, Jimei University, Xiamen, China; 2 Department of Kinesiology & Biomechanical Engineering Doctoral Program, Boise State University, Boise, USA

**Keywords:** Postural automaticity, Neural processing, Muscle response, Aging

## Abstract

The purpose of this study was to investigate the effects of dual-task standing on postural-related muscle activity and cortical activation in both young and older groups. Fourteen older adults and thirteen young adults were recruited. Participants performed single-task and dual-task standing. The surface electromyographic signals of tibialis anterior, solus, rectus femoris, and biceps femoris at left and right sides were recorded. Simultaneously, cortical activation in the dorsolateral prefrontal and motor cortices were measured. Two-way MANOVAs with repeated measures and Pearson correlation analyses were employed for the statistical analysis. Our results indicated that only the older group presented greater right (*p* = .002) and left (*p* = .003) ankle muscle co-activation index, and greater cortical activation in the dorsolateral prefrontal cortex (*p* < .001), premotor motor cortex (*p* = .011), supplementary motor area (*p* = .043), and primary motor cortex (*p* = .028) in the left hemisphere during dual-task compared to single-task standing. Additionally, the older group showed negative correlations, whereas the young group showed positive correlations between cortical activation and average linear envelope of muscle activity during the single-task standing. Furthermore, the older group showed more significant positive correlations between cortical activation and average linear envelope of muscle activity than the young group during dual-task standing. These observations suggest that age-related overactivation of the prefrontal-motor cortex may lead to redundant ankle joint muscle response during dual-task standing.

**Supplementary Information:**

The online version contains supplementary material available at 10.1007/s10548-025-01137-8.

## Introduction

Standing is one of the most important functional activities in daily life. Automaticity, a hallmark of healthy control of standing, refers to the nervous system’s ability to coordinate postural stability in response to complex environments or challenging tasks with minimal use of attention-demanding executive control resources (Schneider and Chein 2003; Clark 2015). The primary central nervous system circuits relevant to postural automaticity may be in the subcortical regions, such as the brainstem, cerebellum, and spinal cord (Horak and Diener 1994; Lewko 1996; Takakusaki et al. 2017). These regions are responsible for programmed neuromuscular coordination and activation patterns in upright standing (Horak and Diener 1994; Lewko 1996; Takakusaki et al. 2017). Individuals are generally unaware of the adjustments in postural-related muscle activity and generate appropriate muscle responses to maintain an upright posture (Takakusaki et al. 2017). With aging, however, the reduction in functional specialization of brain regions shifts from automatic to controlled processing of posture (Laughton et al. 2003; Sullivan et al. 2009; Papegaaij et al. 2014; Clark 2015), which is associated with high risk of falls (Muir-Hunter and Wittwer 2016; Bergen et al. 2021). Controlled processing of posture may be predominantly subject to cortical control, which voluntarily influences muscle responses to counter disturbances that endanger postural stability (Loehrer et al. 2016; Spedden et al. 2020). Consequently, a research question arises as to whether changes in cortical activation would generate appropriate muscle responses to a challenging cognitive task during upright standing among older adults.

The most widely used approach to measure the balance between automatic and controlled processing of posture is the assessment of dual-task paradigm (Fujita et al. 2016; Rosso et al. 2017; Marusic et al. 2019; St-Amant et al. 2020; St George et al. 2021). Prior works have shown that older adults recruit additional attention-demanding executive control resources in response to a secondary cognitive task, as evidenced by higher prefrontal cortex (PFC) activation in the older group compared to the young group during dual-task standing (Rosso et al. 2017; St George et al. 2021). Moreover, motor commands originating from the PFC to the motor cortex may play an important role in modulating postural-related muscle contractions through the descending pathway (Baudry 2016; Takakusaki et al. 2016, 2017). Therefore, many studies also investigated the activation of motor cortex in both young and older adults during dual-task paradigms (Fujita et al. 2016; Stuart et al. 2019; Kim et al. 2021). Increase activation of the motor cortex has been demonstrated as a general response in both young and older adults to cognitive tasks (Fujita et al. 2016; Stuart et al. 2019; Kim et al. 2021). For instance, young adults showed greater supplementary motor area (SMA) activation during dual-task compared to one leg single-task standing (Fujita et al. 2016). Additionally, both older and young adults presented greater bilateral SMA, bilateral premotor cortex (PMC), and bilateral primary motor cortex (M1) activation during dual-task compared to single-task walking (Stuart et al. 2019). Another study indicated that older adults presented greater bilateral PFC, SMA, bilateral PMC, and right M1 during dual-task compared to single-task walking (Kim et al. 2021). However, older adults may present greater activation of motor cortex in response to secondary cognitive task compared to young adults. Prior evidence indicated that older adults showed greater right SMA activation compared to young adults in both weight-shifting task and weight-shifting task with serial subtraction (De Rond et al. 2021). This “overactivation” in the PFC and SMA may reflect age-related neural dysfunction since older adults fail to preserve task or balance performance in response to the secondary cognitive task (Rosso et al. 2017; De Rond et al. 2021; St George et al. 2021). Therefore, in the current study, investigating the activation of the PFC and motor cortex is crucial to address the mentioned research question.

On the other hand, the muscles in the shank and thigh are typically recorded to investigate muscle response contributing to postural control (Laughton et al. 2003; Donath et al. 2016; Vette et al. 2017). Older adults demonstrated worse balance performance compared to young adults during single-task standing, which may be associated with increased muscle activity of tibialis anterior (TA), triceps surae, vastus lateralis (VL), or biceps femoris (BF), and muscle co-activation index of TA-SOL or VL-BF (Laughton et al. 2003; Donath et al. 2016; Vette et al. 2017). Additionally, studies have demonstrated that older adults had greater muscle activity of TA and triceps surae, and worse balance performance compared to young adults during dual-task standing (Melzer et al. 2001; Simoneau et al. 2008). In general, increased muscle activity in response to the secondary cognitive task in older adults aims to adopt a strategy of increased co-contraction of agonist-antagonist muscles for safer postural control (Ruffieux et al. 2015; Rubega et al. 2021). Interestingly, if increased agonist muscle activity coincides with decreased antagonist muscle activity in postural control, it may lead to an increased co-activation index and result in greater deviation from the body’s equilibrium point (Tanabe et al. 2017; Latash 2018). Consequently, older adults could present worse balance performance due to poor agonist-antagonist muscle coordination. However, there is very limited scientific evidence investigating age-related differences in agonist–antagonist muscle coordination, as further indicated by muscle activity and co-activation index, in response to secondary cognitive tasks.

The purpose of this study was to examine dual-task effects on postural-related muscles, including average linear envelope of muscle activity, co-activation index, and agonist-antagonist muscles coordination, and cortical activity in the PFC and motor cortex in both young and older groups. Additionally, we aimed to identify the potential relationship between the average linear envelope of muscle activity and cortical activation within these groups. We hypothesis that the older group could present a greater average linear envelope of muscle activity, greater ankle muscle co-activation index, smaller agonist-antagonist muscles coordination, and higher cortical activation in PFC and motor cortex during dual-task compared to single-task standing, while the young group could show no significant difference between single-task and dual-task standing. We also expected that greater cortical activation in PFC and motor cortex in older adults would relate to greater average linear envelope of muscle activity during dual-task standing.

## Methods

### Participants

A F-test power analysis was conducted using the statistical test of MONOVA with repeated measure (within-between interaction) in the G*Power software (Version 3.1) to estimate the minimal sample size. Based on previous studies, the size of difference in cortical activation between single-task and dual-task paradigm in older adults presented moderate to strong (Salzman et al. 2021; Pan and Zhang 2024). Therefore, the effect size was set at 0.65. Other Parameters set included the α level = 0.05, statistical power = 0.80 (Maxwell et al. 2008), number of groups = 2, and number of measurements = 2, yielding a minimum estimated sample size of 21 (11 participants per group).

We recruited twenty-seven healthy, right-leg dominant adults (13 young adults and 14 older adults) for the study. The young group was recruited from a local university, while the older group was recruited from local communities. The inclusion criteria were: (1) age range from 19 to 30 for young adults; (2) age range from 60 to 75 for older adults; (3) ability to stand without an assistance for 60 s; (4) no history of a lower limb surgery in the last two years; (5) no history of a diagnosis of neurological diseases, such as mild cognitive impairment, permanent memory loss, stroke, Parkinson’s disease, or brain tumors; (6) Mini-Mental State Examination (MMSE) score > 24; and (7) no history of drug and alcohol abuse. The study was conducted in accordance with the Declaration of Helsinki and was approved by the Ethics Committee of the Boise State University (NO. 186-MED23-007). Each participant signed an informed consent form before testing.

### Procedures

Prior to data collection, participants’ body height, body mass, and head circumference were measured. Meanwhile, our experimental operator briefly introduced the test procedure to each participant. Participants were instructed to stand still (single-task) or stand still while simultaneously subtracting 7 from a random 3-digit number (dual-task). Participants gazed at a stationary white “cross” target (2.5 × 2.5 cm) in the center of a 42-inch TV screen with a black background placed 1 m away at eye level (Watanabe et al. 2018). In addition, participants stood with one foot on a force plate, with their feet parallel and 15 cm apart (Watanabe et al. 2018). The order of tasks was fixed: (1) single-task standing, then (2) dual-task standing. Each task started with 10 s of quiet standing (baseline) upon hearing “stand still and look straight ahead” by our experimental operator (Menant et al. 2020). Then, the instruction “go” or “a given 3-digit number” cued participants to perform a balance task for 30 s, followed by the instruction “stop” at the end of the task. Participants were instructed to verbalize their answers at a preferred volume and to concentrate on maximizing the accuracy of their calculations. Each condition was repeated twice.

### Instruments

**Fig. 1:**
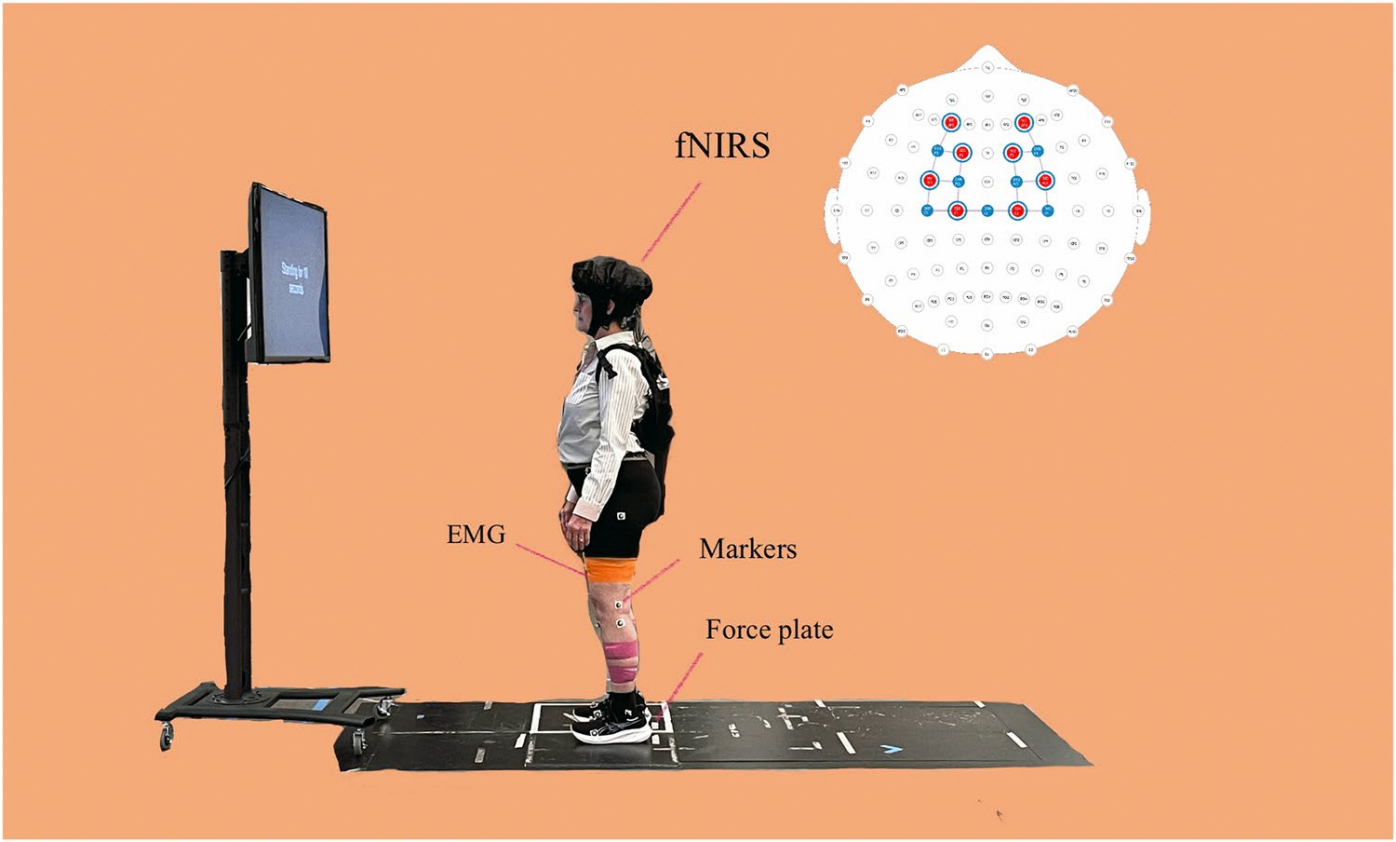
The setup of experiment. For the fNIRS optode montage, solid red circles represent the location of sources, solid blue circles represent the location of detectors, and hollow circles represent the location of short distance detectors

Figure 1 illustrates the experimental setup. The Delsys electromyography (EMG) system (Delsys, Boston, MA, USA) was used to record muscle activity at the sampling rate of 2000 Hz. Prior to placing the surface EMG sensors, the skin was shaved with a razor, wiped with an alcohol pad, and thoroughly air-dried. Eight surface EMG sensors were placed as close to the longitudinal midline of the muscle belly as possible and in the portion of the muscle belly with maximal mass on the tibialis anterior (TA) solus (SOL), rectus femoris (RF), and biceps femoris (BF) on both the left and right sides. These surface EMG sensors were wrapped with an elastic bandage to minimize motion artifacts. Additionally, twenty-six 14 mm reflective markers were attached on the lateral/medial prominence of the lateral femoral epicondyle, proximal tip of the head of the fibula, anterior border of the tibial tuberosity, lateral/medial prominence of the lateral malleolus, dorsal margin of the first, second and fifth metatarsal head and heel. The ten-camera motion capture system (Vantage 6, Vicon Inc., Oxford, UK) was used to record the time-position of each marker at sampling rate of 100 Hz. Meanwhile, two embedded force plates (OR6, AMTI, MA, USA) were used to record three orthogonal forces and free moments from each foot at a sampling rate of 2000 Hz. All these devices were synchronized through the Vicon Lock, thereby can be simultaneously recorded by the Nexus software (Vicon Inc., Oxford, UK).

In this study, change in cortical hemoglobin concentration were measured using the functional near-infrared spectroscopy (fNIR) system (NIRSPORT2, NIRx, Berlin, Germany). The system utilizes near-infrared light (8 LED sources and 8 detectors) transmitted at wavelengths of 760 and 850 nm with a sampling rate of 10.2 Hz. The source-detector consisted of 18 long-distance channels with a distance approximately 30 mm. Additionally, to minimize noise, eight short detectors were clipped under each LED source to record extracortical signals. These short-distance channels had an 8 mm source-detectors separation. These near-infrared lights were placed on a lightweight NIRx cap according to the 10–10 international system. The size of lightweight NIRx cap was chosen based on our participants’ head circumference. During data collection, an opaque black cap was used to cover the sources and detectors to reduce interference from external light sources. The NIRx Cap system covered the dorsolateral prefrontal cortex (DLPFC) and premotor cortex (PMC), supplement motor area (SMA), and primary motor cortex (M1) at both brain hemispheres based on the Brodmann area atlas (Zimeo Morais et al. 2018). The fNIRS system was also synchronized with the Nexus software through the PsychoPy program (v2023.2.1, Open Science Tools Ltd., UK).

### Data Analysis

The primary outcomes, including magnitude of EMG activity, ankle joint muscles co-activation index, and EMG-EMG coordination, were calculated using custom-made algorithms in MATLAB (R2023a, The Mathworks, Natick, USA). Raw EMG signals were removed the outliers using the generalized Studentized deviate test (Vera et al. 2013). After removing possible outliers, the EMG singles were filtered by a band reject filter of 50 Hz, followed by a 20 Hz high-pass and a 400 Hz low-pass fourth-order Butterworth filter (Kurz et al. 2012; Donath et al. 2015). Then, the EMG singles were full wave rectified and linear enveloped using another low-pass fourth-order Butterworth filter with a cut-off frequency of 8 Hz (Akl et al. 2021). The resulting linear envelops were normalized to their mean maximal muscle activity during single-task standing (Halaki and Ginn 2012).

For the magnitude of EMG activity, the average linear envelope of normalized muscle activity was calculated for the TA (TA__R_ & TA__L_), SOL (SOL__R_ & SOL__L_), RF (RF__R_ & RF__L_) and BF (BF__R_ & BF__L_) on both the right and left sides (Murillo et al. 2012). For the muscle co-activation index (CAI), the integral of linear envelope of normalized muscle activity was considered for further analysis (Maktouf et al. 2020). The muscle CAI was calculated for the SOL and TA (CAI__R_LEG_ & CAI__L_LEG_; see Eq. 1) or BF and RF (CAI__R_TH_ & CAI__L_TH_; see Eq. 2) on both sides, as the following formula:

1 $$\:CAI=\:\frac{2\times\:{\int\:}_{{t}_{i}}^{{t}_{f}}TA\:\left(t\right)dt}{{\int\:}_{{t}_{i}}^{{t}_{f}}\left(TA+SOL\right)\left(t\right)dt}$$

2 $$\:CAI=\:\frac{2\times\:{\int\:}_{{t}_{i}}^{{t}_{f}}RF\:\left(t\right)dt}{{\int\:}_{{t}_{i}}^{{t}_{f}}\left(RF+BF\right)\left(t\right)dt}$$

The $\:{t}_{i}$ and $\:{t}_{f}$ mean the onset and ending point of the standing task. This equation assumes TA and RF as the antagonist muscles (Donath et al. 2015). The CAI ranges from 0 (no co-activation) to 200 (maxima co-activation) as the antagonist muscle contribution to the total activity of agonist and antagonist muscles during standing.

The EMG-EMG coordination was the cosine of the angle between resulting linear envelops of TA and SOL (COOR__R_LEG_ & C_OOR_L_LEG_; see Eq. 3) or RF and BF (COOR__R_TH_ & C_OOR_L_TH_; see Eq. 4) on both sides and calculated, as the following formula (Poston et al. 2010):

3 $$\:cos\theta\:=\frac{{\sum\:}_{i=1}^{n}{TA}_{i}\bullet\:{SOL}_{i}}{\sqrt{\sum\:_{i=1}^{n}{{TA}_{i}}^{2}}\sqrt{\sum\:_{i=1}^{n}{{SOL}_{i}}^{2}}}$$

4 $$\:cos\theta\:=\frac{{\sum\:}_{i=1}^{n}{RF}_{i}\bullet\:{BF}_{i}}{\sqrt{\sum\:_{i=1}^{n}{{RF}_{i}}^{2}}\sqrt{\sum\:_{i=1}^{n}{{BF}_{i}}^{2}}}$$

The $\:i$ means the $\:ith$ EMG activity from onset to the ending point of the standing task. The $\:n$ means the number of data points. The range of possible values (0–1) for the cosine of the angle between pairs of agonist and antagonist muscles is used to quantify the degree of similarity in their spatial orientation where the 0 indicates dissimilarity and the 1 indicates similarity.

The cortical hemoglobin concentration signal was pre-processed using the Homer 3 toolbox in MATLAB (R2023a, The Mathworks, Natick, USA), following the distinct steps outlined in the previous studies (Hoang et al. 2022; Bonnal et al. 2023):

1. Identification of channels’ quality: The coefficients of variation (CV, (standard deviation/mean)*100) of each channel for each participant was calculated (Piper et al. 2014; Zimeo Morais et al. 2018). Channels with a CV > 15% were excluded from the further data analysis since signals include physical artifacts (e.g. motion-induced instabilities of the coupling efficiency at the tissue-optical interfaces) and physiological artifacts (e.g. blood-pressure-induced hemodynamics) (Zimeo Morais et al. 2018). The function hmr_PruneChannels was used (SNRthresh = 6).
2. Optical density conversion: Raw data was converted to optical density data. The function hmr_Ientesity2OD was used.
3. Identification of motion artifacts: The signals were considered as motion artifacts if the signals is larger than 5 times of standard deviation or the magnitude of standard deviation is more than 0.5 during a 0.5 s period. The function hmr_MotionArtifcatByChannel was used (tMotion = 0.5, tMask = 1, STDEVthresh = 5.0. & AMPthresh = 0.50).
4. Motion artifacts correction: The signals marked as the motion artifacts were corrected with principal component analysis and recursive filtering. The function hmrMotionCorrectPCArecurese was used (nSV = 0.97). Then, the signals were further corrected with wavelet-based filter. The function hmr_MotionCorrectWavelet was used (iqr = 1.5).
5. Physiological noise correction: Attenuation of heartbeat, respiration, and blood pressure singles were performed by the band-pass filter. The function hmr_BadnpssFilt was used (hpf = 0.01 & lpf = 0.14).
6. Hemoglobin concentration conversion: The optical density data was converted to concentration change in hemoglobin using the modified Beer-Lamber law using function hmrR_OD2Conc. The age-dependent differential path length factor value was calculated by the formula 5.11 + 0.106 × Age^0.723^ for the 760 nm wavelength and 4.67 + 0.062 × Age^0.819^ for the 840 nm wavelength (Duncan et al. 1996).
7. Short channels regression: Attenuation of systemic physiological artifacts in fNIRS was solved by a general linear deconvolution model. The function hmrR_GLM was used (trange = [−5 30], glmSolveMethod = 1, idxBasis = 1, paramsBasis = [0.5 0.5], & rhoSD_ssThresh = 15.0).

Finally, HbO_2_ concentration signals were exported to a TXT file. The average cortical activation for each region of interest, including DLPFC__R_, DLPFC__L_, SAM__R_, SMA__L_, PMC__R_, PMC__L_, M1__R_, and M1__L_, was calculated in each condition (see Table 1) (Kotegawa et al. 2020; Kim et al. 2021).

**Table 1:** The regions of interest are based in the brodmann area

Source-Detector	Optodes	Hemisphere	Brodmann area	Cerebral area
1–1	AF4-F4	Right	9	DLPFC
2 − 1	F2-F4	Right	9	DLPFC
2–2	F2-FC2	Right	6	SMA
3 − 1	FC4-F4	Right	6	PMC
3 − 2	FC4-FC2	Right	6	PMC
3–3	FC4-C4	Right	6	PMC
4 − 2	C2-FC2	Right	6	SMA
4 − 3	C2-C4	Right	4	M1
4–4	C2-Cz	Right	4	M1
5–5	AF3-F3	Left	9	DLPFC
6 − 5	F1-F3	Left	9	DLPFC
6–6	F1-FC1	Left	6	SMA
7 − 5	FC3-F3	Left	6	PMC
7 − 6	FC3-FC1	Left	6	PMC
7–7	FC3-C3	Left	6	PMC
8 − 4	C1-Cz	Left	4	M1
8 − 6	C1-FC1	Left	6	SMA
8 − 7	C1-C3	Left	4	M1

The secondary outcomes included conventional CoP outcomes, and right and left ankle joint range of motion and ankle joint stiffness in the sagittal and frontal planes. These secondary outcomes could help explain the altered postural stability in response to the secondary cognitive task in both the young and older groups. The detailed approach to data analysis was described in the Supplementary Appendix.

### Statistical Analysis

There were four sets of dependent variables, including (1) average linear envelope of muscle activity of TA__R_, TA__L_, SOL__R_, SOL__L_, RF__R_, RF__L_, BF__R_, and BF__L_; (2) muscle CAI that include CAI__R_LEG_, CAI__L_LEG_, CAI__R_TH_, and CAI__L_TH_; (3) EMG-EMG coordination that include COOR__R_LEG_, C_OOR_L_LEG_, COOR__R_TH_, and C_OOR_L_TH_; and (4) cortical activation of DLPFC__R_, DLPFC__L_, SAM__R_, SMA__L_, PMC__R_, PMC__L_, M1__R_, and M1__L_. The errors in fNIRS data are not independent across measurement channels (Huppert 2016). Therefore, Shapiro-Wilk’s test was used to assess the normality of each dependent variable, expect for the dependent variables of cortical activation (α = 0.05). Additionally, the multivariate normality and outliers were examined using Mahalanobis’ distance. If the dependent variable showed non-normally distributed, these variables were log10 transformed before statistical analysis.

For the primary outcomes, we performed four two-way MANOVAs with repeated measures (group: between-subject; task: within-subject) to examine the effects of group and task on average linear envelope of muscle activity, muscle CAI, EMG-EMG coordination, and cortical activation, respectively. When a repeated measures MANOVA was significant, follow-up two-way ANOVA with repeated measure tests and pairwise comparisons with Bonferroni adjustments were used to identify significant main and interaction differences. Partial Eta Squared (η²p) was calculated for overall effects and interactions for MANOVAs and ANONAs, where 0.010 ≤ η²*p* ≤.059 as small effect size, 0.060 ≤ η²*p* ≤.014 as medium effect size, and η²*p* >.14 as large effect size (Cohen 2013). Cohen’s d effect size was calculated to interpret the magnitude of specific post hoc comparisons, where 0.20 ≤ Cohen’s d ≤ 0.49 as small effect size, 0.50 ≤ Cohen’s d ≤ 0.80 as medium effect size, and Cohen’s d > 0.80 as large effect size (Cohen 2013).

Pearson correlation analyses were also used to investigate the relationship between average linear envelope of muscle activity and cortical activation in both single-task and dual-task standing. Correlations coefficient (r) in the ranges of 0.30-0.49, 0.50-0.69, and 0.70 or more were considered to represent weak, moderate, and strong correlation, respectively (Moore and Kirkland 2007; Yun et al. 2024). All statistical analyses were conducted using SPSS (26.0, IBM Inc., Chicago, IL, USA). The significance level was set at α = 0.05.

## Results

There were 8 male and 6 female in the older group (age: 66.14 ± 4.04 years; height: 1.73 ± 0.10 m; mass: 72.48 ± 11.37 kg; head circumference: 56.34 ± 0.52 cm; and MMSE: 29.29 ± 0.91). The young group consisted of 7 male and 6 female (age: 22.00 ± 3.34 years; height: 1.75 ± 0.090 m; mass: 75.10 ± 10.70 kg; head circumference: 56.17 ± 0.51 cm; and MMSE: 29.54 ± 0.88). Detailed results of secondary outcomes were described in the Supplementary Appendix.

### Average Linear Envelope of Muscle Activity

There was only a significant task effect (F (8, 18) = 2.996, Wilks’ Lambda = 0.429, *p* =.025, η²*p* =.571) in the average linear envelope of muscle activity. Follow-up ANOVA with repeated measure tests indicated significant task effect in the TA__R_ (F (1, 25) = 13.217, *p* =.001, η²*p* =.346), SOL__R_ (F (1, 25) = 14.197, *p* =.001, η²*p* =.362), RF__R_ (F (1, 25) = 7.038, *p* =.014, η²*p* =.220), BF__R_ (F(1, 25) = 5.075, *p* =.033, η²*p* =.169), TA__L_ (F(1, 25) = 7.334, *p* =.012, η²*p* =.227), SOL__L_ (F(1, 25) = 10.864, *p* =.003, η²*p* =.303), and RF__L_ (F(1, 25) = 4.780, *p* =.038, η²*p* =.161) muscle activities (see Table 2). While dual-task standing presented greater TA__R_, SOL__R_, RF__R_, BF__R_, TA__L_, SOL__L_, and RF__L_ muscle activities compared to single-task standing across the groups (*p* <.05).

**Table 2:** Mean values (± standard deviation) of average linear envelope of muscle activity during both single- and dual-task standing in both the older and young groups

Variables	Single-task		Dual-task	
	Older group	Young group	Older group	Young group
TA__R_ (%) ^T^	49.46 ± 9.95	60.57 ± 6.77	63.04 ± 19.55	69.92 ± 18.51
SOL__R_ (%) ^T^	54.18 ± 8.74	58.05 ± 6.94	75.38 ± 23.67	65.61 ± 20.34
TA__L_ (%) ^T^	52.98 ± 4.36	56.64 ± 3.79	61.79 ± 14.16	64.20 ± 18.64
SOL__L_ (%) ^T^	52.61 ± 4.17	54.20 ± 4.79	66.04 ± 20.42	64.38 ± 15.38
RF__R_ (%) ^T^	54.67 ± 8.32	60.21 ± 5.55	71.01 ± 23.09	69.40 ± 27.10
BF__R_ (%) ^T^	48.89 ± 10.51	51.80 ± 8.82	85.23 ± 78.98	63.70 ± 28.54
RF__L_ (%) ^T^	58.60 ± 5.92	61.85 ± 3.56	81.01 ± 34.27	79.74 ± 60.94
BF__L_ (%) ^T^	46.19 ± 12.99	52.88 ± 7.37	56.86 ± 30.67	59.33 ± 21.79

R means right side; L means left side; TA means tibial anterior; SOL means soleus; RF means rectus femoris; and BF means biceps femoris. ^G^ Indicates a significant group difference in the follow-up ANOVA analysis. ^T^ Indicated a significant task difference in the follow-up ANOVA analysis. ^I^ Indicated a significant interaction difference in the follow-up ANOVA analysis

### Muscle co-activation Index

**Fig. 2:**
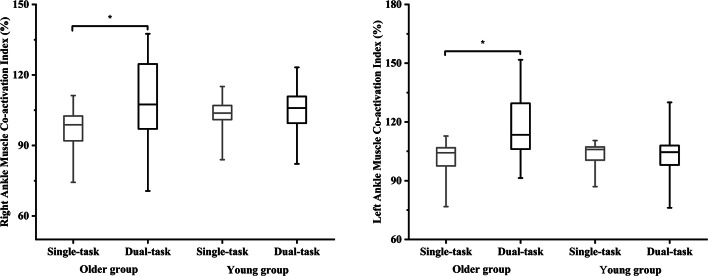
Co-activation index of the right/left tibial anterior and solus muscles in both the young group and older group during the single-task and dual-task standing. * Indicated the observed significant statistical difference between single-task and dual-task standing in older adults

There were significant task effects (F (4, 22) = 3.239, Wilks’ Lambda = 0.629, *p* =.031, η²*p* =.371) and group × task interaction effect (F (2, 23) = 3.485, Wilks’ Lambda = 0.612, *p* =.024, η²*p* =.388) in the muscle CAI. Follow-up ANOVA with repeat measure tests showed significant task effect in the muscle CAI__R_LEG_ (F (1, 25) = 6.804, *p* =.015, η²*p* =.214) and group × task interaction effect in both the muscle CAI__R_LEG_ (F (1, 25) = 5.515, *p* =.044, η²*p* =.153) and CAI__L_LEG_ (F (1, 25) = 6.765, *p* =.015, η²*p* =.213) (see Table 3). While the older group had greater muscle CAI__R_LEG_ (*p* =.002, Cohen’s d = 0.96) and CAI__L_LEG_ (*p* =.003, Cohen’s d = 1.07) during dual-task compared to single-task standing, but there was no significant difference between single-task and dual-task standing in the young group (see Fig. 2).

**Table 3:** Mean values (± standard deviation) of muscle co-activation index during both single- and dual-task standing in both the older and young groups

Variables	Single-task		Dual-task	
	Older group	Young group	Older group	Young group
CAI__R_LEG_ (%) ^T & I^	95.46 ± 11.14	103.03 ± 7.36	109.05 ± 18.76	104.42 ± 10.28
CAI__L_LEG_ (%) ^I^	100.88 ± 9.75	103.55 ± 6.35	115.97 ± 16.40	101.21 ± 16.01
CAI__R_TH_ (%)	105.41 ± 9.94	108.54 ± 9.62	106.03 ± 25.97	104.42 ± 10.28
CAI__L_TH_ (%)	113.67 ± 13.83	109.02 ± 10.16	126.60 ± 23.45	109.70 ± 27.67

R means right side; L means left side; CAI means co-activation index; LEG means the tibial anterior and soleus; and TH means the rectus femoris and biceps femoris. ^G^ Indicates a significant group difference in the follow-up ANOVA analysis. ^T^ Indicated a significant task difference in the follow-up ANOVA analysis. ^I^ Indicated a significant interaction difference in the follow-up ANOVA analysis

### EMG-EMG Coordination

For the EMG-EMG coordination, there was only a significant group effect (F (4, 22) = 3.601, Wilks’ Lambda = 0.604, *p* =.021, η²*p* =.396). Follow-up ANOVA with repeated measure tests observed significant group effects in the COOR__R_LEG_ (F (1, 25) = 4.562, *p* =.043, η²*p* =.154) and COOR__L_LEG_ (F (1, 25) = 5.319, *p* =.030, η²*p* =.175) (see Table 4). While the older group had smaller COOR__R_LEG_ and COOR__L_LEG_ than the young group across the tasks (*p* <.05).

**Table 4:** Mean values (± SD) of EMG-EMG coordination during both single- and dual-task standing in both the older and young groups

Variables	Single-task		Dual-task	
	Older group	Young group	Older group	Young group
COOR__R_LEG_ (%) ^G^	0.95 ± 0.039	0.97 ± 0.014	0.94 ± 0.028	0.96 ± 0.017
COOR__L_LEG_ (%) ^G^	0.95 ± 0.020	0.96 ± 0.013	0.93 ± 0.041	0.96 ± 0.015
COOR__R_TH_ (%)	0.94 ± 0.039	0.96 ± 0.015	0.93 ± 0.045	0.95 ± 0.031
COOR__L_TH_ (%)	0.93 ± 0.057	0.96 ± 0.019	0.88 ± 0.11	0.94 ± 0.028

R means right side; L means left side; COOR means EMG-EMG coordination; LEG means the tibial anterior and soleus; and TH means the rectus femoris and biceps femoris. ^G^ Indicates a significant group difference in the follow-up ANOVA analysis. ^T^ Indicated a significant task difference in the follow-up ANOVA analysis. ^I^ Indicated a significant interaction difference in the follow-up ANOVA analysis

### Cortical Activation

**Fig. 3:**
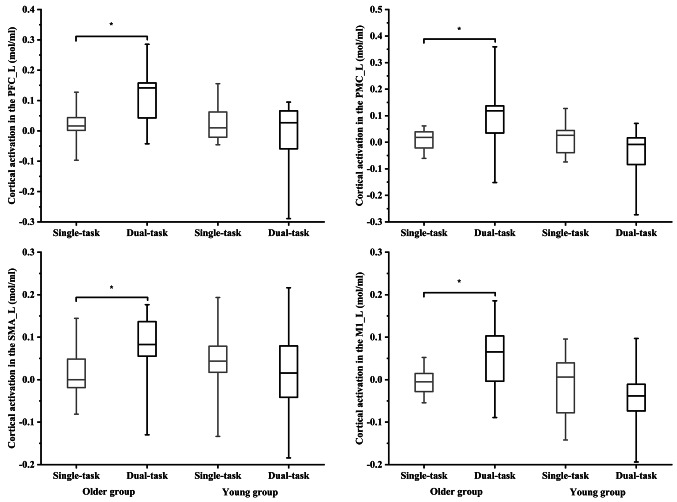
Cortical activation in the left prefrontal cortex (PFC__L_), premotor cortex (PMC__L_), supplementary motor area (SMA__L_), and primary motor cortex (M1__L_) in both the young group and older group during the single-task and dual-task standing. * Indicated the observed significant statistical difference between single-task and dual-task standing in older adults

For the cortical activation, there were significant group effect (F (8, 18) = 4.154, Wilks’ Lambda = 0.351, *p* =.006, η²*p* =.649) and group × task interaction effect (F (8, 18) = 2.595, Wilks’ Lambda = 0.464, *p* =.044, η²*p* =.536). Follow-up tests reported significant group effect in the DLPFC__L_ (F (1, 25) = 5.576, *p* =.026, η²*p* =.182), PMC__L_ (F (1, 25) = 7.416, *p* =.012, η²*p* =.229), and SMA__L_ (F (1, 25) = 6.579, *p* =.017, η²*p* =.208), and group × task interaction effect in the PMC__R_ (F (1, 25) = 5.501, *p* =.027, η²*p* =.180), DLPFC__L_ (F (1, 25) = 16.429, *p* <.001, η²*p* =.397), PMC__L_ (F (1, 25) = 10.094, *p* =.004, η²*p* =.288), SMA__L_ (F (1, 25) = 4.876, *p* =.037, η²*p* =.163), and M1__L_ (F (1, 25) = 4.271, *p* =.049, η²*p* =.146) (see Table 5). Specifically, the young group presented smaller cortical activation in the PMC__R_ during dual-task compared to single-task standing (*p* =.041, Cohen’s d = 0.64), but no significant difference in the older group. Additionally, the older group presented greater cortical activation in the DLPFC__L_ (*p* <.001, Cohen’s d = 1.36), PMC__L_ (*p* =.011, Cohen’s d = 0.92), SMA__L_ (*p* =.043, Cohen’s d = 0.82), and M1__L_ (*p* =.028, Cohen’s d = 0.75) during dual-task compared to single-task standing, but no significant difference in the young group (see Fig. 3).

**Table 5:** Mean values (± SD) of cortical activation during both single- and dual-task standing in both the older and young groups

Variables	Single-task		Dual-task	
	Older group	Young group	Older group	Young group
PFC__R_ (mol/ml)	0.025 ± 0.051	0.039 ± 0.096	0.059 ± 0.11	0.052 ± 0.11
PMC__R_ (mol/ml) ^I^	0.0041 ± 0.041	0.039 ± 0.10	0.039 ± 0.093	−0.028 ± 0.11
SMA__R_ (mol/ml)	0.013 ± 0.047	0.033 ± 0.091	0.023 ± 0.095	−0.019 ± 0.099
M1__R_ (mol/ml)	0.0098 ± 0.045	0.050 ± 0.25	0.047 ± 0.097	0.041 ± 0.20
PFC__L_ (mol/ml) ^G & I^	0.016 ± 0.054	0.027 ± 0.061	0.12 ± 0.094	−0.019 ± 0.11
PMC__L_ (mol/ml) ^G & I^	0.0095 ± 0.035	0.011 ± 0.058	0.091 ± 0.12	−0.044 ± 0.10
SMA__L_ (mol/ml) ^I^	0.0071 ± 0.059	0.040 ± 0.089	0.071 ± 0.093	0.0091 ± 0.11
M1__L_ (mol/ml) ^G & I^	−0.017 ± 0.063	−0.013 ± 0.071	0.11 ± 0.23	−0.048 ± 0.073

R means right side; L means left side; PFC means prefrontal cortex, PMC means premotor cortex, SMA means supplementary motor area, M1 means primary motor cortex. ^G^ Indicates a significant group difference in the follow-up ANOVA analysis. ^T^ Indicated a significant task difference in the follow-up ANOVA analysis. ^I^ Indicated a significant interaction difference in the follow-up ANOVA analysis

### Correlation between Neuromuscular Features and Cortical Activation

In the older group, during single-task standing, we detected a moderate negative correlation between cortical activation in the DLPFC__L_ and muscle activity of TA__L_ (*r* = −.649, *p* =.012) and a strong negative correlation between cortical activation in the SMA__L_ and muscle activity of SOL__R_ (*r* = −.803, *p* =.001). During dual-task standing, we observed a moderate positive correlation between cortical activation in the PMC__R_ and muscle activity of TA__L_ (*r* =.581, *p* =.029), cortical activation in the SMA__R_ and muscle activity of SOL__L_ (*r* =.550, *p* =.041), cortical activation in the M1__R_ and muscle activity of TA__R_ (*r* =.546, *p* =.043), cortical activation in the DLPFC__L_ and muscle activity of TA__R_ (*r* =.622, *p* =.018), and cortical activation in the M1__L_ and muscle activity of TA__R_ (*r* =.608, *p* =.021), SOL__R_ (*r* =.579, *p* =.030), and RF__R_ (*r* =.562, *p* =.036). Additionally, there was a strong positive correlation between cortical activation in the M1__L_ and muscle activity of RF__L_ (*r* =.905, *p* <.001) during dual-task standing.

In the young adults, during single-task standing, we only detected a strong positive correlation between cortical activation in the M1__L_ and muscle activity of SOL__R_ (*r* =.702, *p* =.008), and a moderate positive correlation between cortical activation in the M1__L_ and muscle activity of BF__L_ (*r* =.604, *p* =.029). During dual-task standing, we observed a moderate positive correlation between cortical activation in the PFC__R_ and muscle activity of SOL__L_ (*r* =.554, *p* =.049), and cortical activation in the SMA__L_ and muscle activity of SOL__L_ (*r* =.574, *p* =.040).

## Discussion

The purpose of this study was to investigate the effect of dual-task standing on EMG activity in postural-related muscles, as assessed by average linear envelope of muscle activity, muscle CAI, and EMG-EMG coordination, and cortical activation in DLPFC and motor cortex between young and older adults. Specifically, we expected significant group × task interaction on these outcomes. Our results indicated that (1) the older group showed greater right and left ankle muscle CAI, and greater cortical activation in the DLPFC__L_, PMC__L_, SMA__L_, and M1__L_ during dual-task compared to single-task standing, but no significant difference between single-task and dual-task standing in the young group. (2) The older group had smaller right and left TA-SOL coordination than the young group, regardless of task. (3) Both the young and older groups presented greater muscle activity in the right (TA, SOL, RF, and BF) and left (TA, SOL, and RF) sides during dual-task compared to single-task standing. Additionally, we identified the relationship between average linear envelope of muscle activity and cortical activation in both young and older adults during dual-task standing. We expected strong correlations between cortical activation and average linear envelope of muscle activity in older adults during dual-task standing. Our results further indicated that (1) during single-task standing, greater cortical activation was related to a decrease in average linear envelope of muscle activity in the older group (negative correlation), whereas greater cortical activation was related to an increase in average linear envelope of muscle activity in the young group (positive correlation). (2) During dual-task standing, the older group showed more significant positive correlations between cortical activation and average linear envelope of muscle activity than the young group. These observations suggest that the age-related loss of postural automaticity may be associated with the over-activation in the left prefrontal and motor cortices, which can no longer produce an appropriate postural-related muscle response during dual-task standing.

Prior works suggested that greater cortical activation in the DLPFC and motor cortex, combined with worse balance performance in response to the secondary cognitive task in older adults, would reflect a loss of postural automaticity in older adults (Rosso et al. 2017; De Rond et al. 2021; St George et al. 2021) due to hemispheric asymmetry reduction (Cabeza 2002) or less specificity of neural processing (Cabeza 2002; Shokri-Kojori et al. 2012; Alain et al. 2022). Our results support these works, as older adults present greater cortical activation in the DLPFC__L_, worse balance performance, greater ankle joint ROM, and smaller ankle joint stiffness (see Supplementary Appendix) during dual-task compared to single-task standing. Moreover, our results further indicate that older adults with a loss of postural automaticity may rely more on the prefrontal-motor cortex circuit, as indicated by greater cortical activation in the PMC__L_, SMA__L_, and M1__L_ during dual-task compared to single-task standing (Li et al. 2015; Wang and Sun 2024). Specifically, the attention-demanding signal from the PFC may trigger the PMC and SMA to produce motor programs (Ghez and Krakauer 2000; Takakusaki et al. 2017; Palmer et al. 2021). These motor programs are then sent to the M1, enabling controlled processing of postural-related muscles (Ghez and Krakauer 2000; Takakusaki et al. 2017; Palmer et al. 2021). When older adults have deficits in postural automaticity, therefore, increased activation in the PMC and SMA may play an important role in promoting and suppressing activation in the M1 (Grefkes et al. 2008; Spedden et al. 2020). Scientific evidence has suggested that age-related reduction in the ability to regulate M1 inhibition is associated with poor task performance, as shown in a transcranial magnetic stimulation study (Corp et al. 2018). Additionally, stronger PMC-M1 coupling was associated with better force precision in older adults during a unilateral dorsiflexion force-tracking task (Spedden et al. 2020). Therefore, our results may suggest that over-activation of the DLPFC__L_, PMC__L_, SMA__L_ fails to suppress the M1__L_ activation, resulting in worse balance performance in older adults during dual-task standing. However, a limitation of our study is that we did not examine the function connectivity among these cortical regions. Future working comparing coupling parameters is necessary to clarify how aging affects the prefrontal-motor network in response to the secondary cognitive task.

Heightened activity in the agonist muscle could decrease the inhibition of the activity in the paired antagonist muscle due to age-related dysfunction in contribution of spinal and corticospinal pathways to control posture, especially in response to the secondary cognitive task (Hortobágyi et al. 2006; Papegaaij et al. 2014; Ruffieux et al. 2015). Therefore, we expected that only older adults presented greater muscle activity in both agonist and antagonist muscles during dual-task compared to single-task standing. However, our observations reported greater right (TA, SOL, RF, and BF) and left (TA, SOL, and RF) muscle activity across the groups during dual-task compared to single-task standing. Interestingly, our current observations in young adults are consistent with some previous studies (Choi et al. 2011; Hill et al. 2019). For instance, prior study indicated that young adults showed greater muscle activity of TA during dual-task (choice reaction task) compared to single-task standing (standing on one force plate) (Choi et al. 2011; Hill et al. 2019). Another study reported that dual-task (visual Stroop task) standing had greater muscle activity of TA and vastus medialis compared to quite standing (three different surfaces) (Choi et al. 2011; Hill et al. 2019). These observations suggest that increased cognitive demand also influences the control of postural-related muscles in young adults (Choi et al. 2011; Hill et al. 2019), which may also lead to greater postural sway in young adults during dual-task compared to single-task standing according to our results (see Supplementary Appendix). Additionally, our results of postural-related muscle activity between single-task and dual-task standing are inconsistent with some previous studies (Simoneau et al. 2008; Makizako et al. 2013; Saraiva et al. 2023, 2024). For example, prior works reported that young or older adults present smaller TA (Rankin et al. 2000; Simoneau et al. 2008; Saraiva et al. 2023, 2024), triceps surae (Simoneau et al. 2008; Makizako et al. 2013; Saraiva et al. 2023, 2024), RF (Saraiva et al. 2023, 2024), and BF (Saraiva et al. 2023, 2024) muscle activity during dual-task (verbal fluency task, subtraction by threes, visual–spatial memory task) compared to single-task standing (quite standing, standing with holding a glass full of sand, tandem standing or standing on a compliant foam surface). The possible reason could be explained by the different secondary cognitive tasks and normalization techniques implemented (normalized by maximal muscle activity of single-task standing vs. peak maximal voluntary contraction) for EMG activity in our study compared to prior studies. Therefore, future work comparing EMG activity under different task conditions is necessary to clarify this issue.

Additionally, heightened TA-SOL muscle CAI in response to the secondary cognitive task is observed in older adults in the current study. Based on previous review articles, these results could be explained by the neural adaptation with the stiffer ankle joints as the strategy to maintain postural stability in older adults during dual-task standing (Ruffieux et al. 2015; Rubega et al. 2021; Kim and Chou 2022). The reason is that increased muscle CAI is associated with reducing ankle joint rotational degrees of freedom during upright stance due to loss of postural flexibility (Latash 2018; Rubega et al. 2021). From our secondary outcomes, however, our older group presented worse balance performance assessed by the conventional CoP outcomes, greater ankle joint ROM, and smaller ankle joint stiffness in both the sagittal and frontal planes during dual-task compared to single-task standing (see Supplementary Appendix). Taken together, these observations may suggest that increased ankle muscle CAI in response to the secondary task in older adults is associated with an “aging-related degeneration of postural control”, indicating a high risk of falls (Latash 2018; Falk et al. 2022). In agreement with prior works indicating that increased ankle muscle CAI is associated with greater postural sway during upright stance (Laughton et al. 2003; Donath et al. 2016; Vette et al. 2017). Several review articles have argued that the interpretation of muscle CAI should be discussed within the framework of the equilibrium-point hypothesis (Latash 2010, 2018; Falk et al. 2022). Specifically, motor synergies, the coordinated activation of agonist-antagonist muscle groups, could describe how these muscle groups are organized to ensure stable and adaptable motor performance (Latash 2010). Our results further showed that the older group had smaller right and left TA-SOL coordination than the young group, regardless of task. Smaller EMG-EMG coordination reflects a greater magnitude of dissimilarity in the degree of coordination (Poston et al. 2010). We speculate that increased antagonist muscle activity in response to the secondary cognitive task occurring simultaneously with decreased activity in the agonist muscle could produce worse right and left TA-SOL coordination (Tanabe et al. 2017; Latash 2018), resulting in body position far away from body’s equilibrium point. Therefore, our observations may further suggest age-related deficits in utilizing the overactivation of prefrontal-motor cortex to assist in coordination between agonist-antagonist muscles combinations and modulating postural control. The reason is that the motor commands in older adults may over suppress the redundant degrees of freedom available in the musculoskeletal system to ensure both stability and adaptability in motor tasks (Latash 2010)​.

Generating appropriate muscle responses with minimal use of cortical resources would reflect a healthy control of posture (Schneider and Chein 2003; Clark 2015). When individuals present loss of postural automaticity, the additional electrical impulses from the cortical regions may descend to the brainstem and spinal motor centers in the corticospinal tract, and then innervate the lateral and ventral columns of the spinal cord to serve the increased voluntary movement of contralateral limbs (Javed et al. 2018), and increased axial and proximal muscle activity involved in postural control (Purves et al. 2019), respectively. For instance, prior works reported that the transcranial magnetic stimulations of the motor cortex produced increased evoked motor-evoked potentials in the SOL and TA during unsupported standing compared to supported standing/sitting (Obata et al. 2009; Tokuno et al. 2009). Compared to young adults, however, older adults present greater amplitude of motor-evoked potentials in the SOL during standing (Baudry et al. 2015; Papegaaij et al. 2016). Hence, we expected to observe a more significant positive correlation between cortical activation of DLPFC and motor cortex and average linear envelope of muscle activity in older adults compared to young adults during dual-task standing. Our observations are in agreement this hypothesis. Especially, the increased cortical activity in the M1__L_ was associated with greater muscle activity of TA__R_, SOL__R_, RF__R_, and RF__L_, while the DLPFC__L_ and M1__R_ were associated with greater muscle activity of TA__R_ in older adults during dual-task standing. However, in the young group, the increased cortical activity in DLPFC__R_ and SMA__L_ were only associated with greater muscle activity of SOL__L_ during dual-task standing, leading to the body returning to its equilibrium point. These observations would further explain the age-related loss of postural automaticity due to redundant adjustments of the postural-related muscles resulting in postural instability, as the overactivation of PFC and motor cortex in response secondary cognitive task (Corp et al. 2018). Interestingly, during single-task standing, the older group presented a negative correlation between cortical activation in the DLPFC__L_ or SMA__L_ and muscle activity of TA__L_ or SOL__R_, respectively. However, the young group showed a positive correlation between cortical activation in the M1__L_ and muscle activity of SOL__R_ and BF__L_. Given these results, we speculate that the increased cortical activation may play an important role in inhibiting muscle activity in older adults during single-task standing. This statement is supported by previous works (Opie and Semmler 2016; Papegaaij et al. 2016). For instance, stimulating the M1 using transcranial magnetic stimulations during standing indicated that age-related reduction by 30% in the short-interval intracortical inhibition for TA muscles, but no significant difference in TA muscle activity between young and older adults (Papegaaij et al. 2016). Therefore, older adults may rely on PFC and SMA to compensate for the reduction in ability to regulate M1 inhibition, leading to appropriate muscle response during single-task standing. During dual-task standing, overactivation of the PFC and SMA in older adults may fail to overcome the deficits in M1 inhibition due to age-related loss of postural automaticity.

There are several limitations in the current study. First, the small sample size may limit the generalizability of the observations. Second, our study design utilized quiet standing as the primary motor task, which makes it difficult to identify the agonist and antagonist muscles. This is because quite standing present small deviation of postural sway, thereby constraining our ability to discover differences in muscle activity in response to the secondary cognitive task between young and older adults. Further study should consider alternative primary motor tasks that could clear the function of muscles during movement. Third, the strength of our interpretation, which increased cortical activation in the DLPFC, PMC, and SMA fail to promote the ability to regulate M1 inhibition in older adults, may not be strong enough. This is because our interpretation is based on cortical activation measured by fNIRS and muscle activity measured by EMG, which do not allow for establishing a point-to-point relationship from the cortex to muscle. Therefore, future study is necessary to determine the directed connectivity between M1 inhibition ability and muscle response to the secondary cognitive task in young and older adults using the transcranial magnetic stimulation.

## Conclusion

The current study reported that older adults presented increased right and left TA-SOL muscle CAI in response to the secondary cognitive task, suggesting that they may adopt a “safety strategy” to maintain postural stability. Surprisingly, the “safety strategy” in our older adults did not positively contribute to their postural control ability, as they showed smaller right and left TA-SOL coordination compared to young adults across the tasks. In addition, our older group also presented increased cortical activation in the left DLPFC and motor cortices in response to the secondary cognitive task. Moreover, there is more significant positive correlation between cortical activation and muscle activity in the older group compared to the young group under the dual-task standing. Taken together, age-related recruitment of additional neural resources from the prefrontal-motor cortex may be associated with redundant ankle joint muscle response during dual-task standing.

## Supplementary Information

Below is the link to the electronic supplementary material.

Supplementary Material 1

## Data Availability

The data used during the current study is available from the corresponding author on reasonable request.

## Declarations

### Supplementary Appendix

The data analysis and results of secondary outcomes were shown in the Supplementary Appendix.
