# Supplementary material for: Effect of Dual-task Standing on prefrontal-motor Cortex Activation and postural-related Muscle Activity between Young and Older Adults
Source: Brain Topogr. 2025 Sep 11;38(6):64. doi: 10.1007/s10548-025-01137-8 (PMC12426098; doi:10.1007/s10548-025-01137-8)
Supplement: Supplementary file 1 — Supplementary Material 1 [file 10548_2025_1137_MOESM1_ESM.docx]

**Methods**

***Data analysis***

The secondary outcomes, including conventional center of pressure (CoP) outcomes from each foot and net CoP, were calculated using custom algorithms in MATLAB (R2023a, The Mathworks, Natick, USA). The data from two force plates were filtered with a low-pass, zero-lag, fourth-order Butterworth filter with cutoff frequencies of 10 Hz (Duarte and Zatsiorsky 2002; Menegoni et al. 2009). Since two force plates were used in the current study, the net CoP (see Equation A1) was calculated (Winter et al. 1996).

${CoP}_{net}\left( i \right)= {CoP}_{L}\left( i \right)\cdot\frac{R_{VL}(i)}{R_{VL}\left( i \right)+R_{VR}(i)}+{CoP}_{L}\left( i \right)\cdot\frac{R_{VR}(i)}{R_{VL}\left( i \right)+R_{VR}(i)}$ (Equation A1)

The ${CoP}_{L}\left( i \right)$ and ${CoP}_{L}\left( i \right)$ mean the real-time CoP under the left and right limbs, respectively. The $R_{VL}(i)$ and $R_{VR}(i)$ mean the real-time vertical ground reaction force under the left and right limbs, respectively. The standard deviation (SD) of CoP (CoP variability, SD_AP_ & SD_ML_), average velocity (VEL_AP_ & VEL_ML_) for anterior-posterior (AP) and medial-lateral (ML) directions, and the 95% ellipse of sway area (SA) were calculated.

The secondary outcomes also included ankle joint range of motion (ROM) and ankle joint stiffness in the sagittal and frontal planes, which were calculated using Visual3D (C-MOTION, Inc, Germantown, MD, USA). First, the gap-filling of marker trajectory was processed with Nexus software and then exported along with the force plates data for data processing with Visual3D. The marker position data and force plate data were filtered with a low-pass, zero-lag, fourth-order Butterworth filter with cutoff frequencies of 10 Hz (Chua et al. 2014; Oyama et al. 2017) and 10 Hz (Duarte and Zatsiorsky 2002; Menegoni et al. 2009), respectively. Then, the CoP variability was calculated as standard deviation of CoP in the AP and ML direction on the left and right sides. The joint angle in the sagittal and frontal planes was calculated as the coordination of distal segment relative to proximal segment. The ankle joint’s ROM in the sagittal and frontal planes were calculated as the difference between the maximal angle and minimal angle.

In addition, the ankle joint stiffness (see Equation A2) in the sagittal and frontal planes was calculated by fitting a second order model using the formula as follows (Joshi et al. 2021):

$K=\frac{T-I\ddot{\theta}+B\dot{\theta}}{\theta}$ (Equation A2)

While the $T$ means range of ankle joint moment, $I$ means the inertia of foot ($I=M_{foot}\times(L_{foot}\times0.475)^2$), $B$ means viscosity of the ankle joint ($B=T/(\dot{\theta}\times0.0174533)$), $\ddot{\theta}$ means range of ankle joint acceleration, $\dot{\theta}$ means range of ankle joint velocity, and $\theta$means range of ankle joint angle. The range of $T$, $\ddot{\theta}$, $\dot{\theta}$, and $\theta$ were calculated as the difference between maximal and minimal value. $M_{foot}$ means the mass of foot segment. $L_{foot}$ means the length of foot segment.

***Statistical analysis***

For the secondary outcomes, we performed four two-way MANOVAs with repeated measures to examine the effects of group and task on conventional CoP outcomes from each foot, conventional CoP_net_ outcomes, ankle joint ROM, and ankle joint stiffness, respectively. If applied, the two-way ANOVA with repeated measure tests with Bonferroni adjustments were used to identify significant main and interaction differences.

**Results**

***Secondary outcomes***

There were significant group (F_10, 16_ = 3.618, Wilks’ Lambda = 0.307, Partial Eta^2^ = 0.693, *p* = .011) and task (F_10, 16_ = 4.105, Wilks’ Lambda = 0.280, Partial Eta^2^ = 0.720, *p* = .006) effects, and group × task interaction effect (F_10, 16_ = 2.707, Wilks’ Lambda = 0.371, Partial Eta^2^ = 0.629, *p* = .037) in the conventional CoP outcomes from each foot. Follow-up tests indicated significant group and task effects, and group × task interaction effect in the left VEL_AP_ (group effect: F_1, 25_ = 12.877, *p* = .001, task effect: F_1, 25_ = 22.548, *p* < .001, & interaction effect: F_1, 25_ = 5.845, *p* = .019) (see Table A1). Specifically, the left VEL_AP_ (*p* < .001, Cohen’s d = 1.11) presented greater in the older group when performing the dual-task compared to single-task standing, but no significant difference in the young group. Additionally, there was significant group effect in the right VEL_AP_ (F_1, 25_ = 12.109, *p* = .002), right VEL_ML_ (F_1, 25_ = 15.015, *p* = .001), and left VEL_ML_ (F_1, 25_ = 11.182, *p* = .003), and task effect in the right SD_AP_ (F_1, 25_ = 8.415, *p* = .008), left SD_AP_ (F_1, 25_ = 21.473, *p* < .001), right VEL_AP_ (F_1, 25_ = 20.098, *p* < .001), right VEL_ML_ (F_1, 25_ = 21.355, *p* < .001), left VEL_ML_ (F_1, 25_ = 22.453, *p* < .003), right SA (F_1, 25_ = 8.541, *p* = .007), and left SA (F_1, 25_ = 12.234, *p* = .002). While the older group showed greater right VEL_AP_, right VEL_ML_, and left VEL_ML_ than the young group across the tasks (*p* < .05). Also, the dual-task standing had greater right SD_AP_, left SD_AP_, right VEL_AP_, right VEL_ML_, left VEL_ML_, right SA, and left SA than single-task standing across the groups (*p* < .05).

There were significant group (F_5, 21_ = 4.276, Wilks’ Lambda = 0.495, Partial Eta^2^ = 0.505, *p* = .008) and task (F_5, 21_ = 8.798, Wilks’ Lambda = 0.323, Partial Eta^2^ = 0.677, *p* < .001) effects in the conventional CoP outcomes from CoP_net_. Follow-up tests indicated significant group task effect in the SD_ML_ (F_1, 25_ = 7.303, *p* = .012), VEL_AP_ (F_1, 25_ = 16.650, *p* = .001), and VEL_ML_ (F_1, 25_ = 10.313, *p* = .004) and significant task effect in the SD_ML_ (F_1, 25_ = 20.079, *p* < .001), VEL_AP_ (F_1, 25_ = 28.558, *p* < .001), VEL_ML_ (F_1, 25_ = 29.795, *p* < .001), and SA (F_1, 25_ = 10.192, *p* = .004) (see Table A1). While the older group showed greater SD_ML_, VEL_AP_, and VEL_ML_ than the young group across the tasks (*p* < .05). Also, the dual-task standing had greater SD_ML_, VEL_AP_, VEL_ML_, and SA than single-task standing across the groups (*p* < .05).

There was only a significant task (F_4, 21_ = 5.248, Wilks’ Lambda = 0.500, Partial Eta^2^ = 0.371, *p* = .004) effects in the ankle joint ROM. Follow-up tests showed significant task effect in the right and left ankle joint ROM in the sagittal (right: F_1, 25_ = 6.719, *p* = .016 & left: F_1, 25_ = 5.678, *p* = .025) and frontal (right: F_1, 25_ = 14.058, *p* = .001 & left: F_1, 25_ = 11.915, *p* = .002) planes (see Table A1). Specifically, the dual-task standing presented greater right and left ankle joint ROM in the sagittal and frontal planes than dual-task standing across the groups (*p* < .05).

Additionally, there was only a significant task effect (F_4, 21_ = 5.699, Wilks’ Lambda = 0.479, *p* = .003) in the ankle joint stiffness. The follow-up tests displayed significant task effect in the right and left ankle joint stiffness in the sagittal (right: F_1, 25_ = 11.203, *p* = .003 & left: F_1, 25_ = 5.742, *p* = .024) and frontal (right: F_1, 25_ = 16.595, *p* < .001 & left: F_1, 25_ = 19.651, *p* < .001) planes (see Table A1). Specifically, the single-task standing had greater right and left ankle joint stiffness in the sagittal and frontal planes compared to dual-task standing across the groups (*p* < .05).

**Table A1.** Mean values (±SD) of secondary outcomes during both single- and dual-task standing in both the older and young groups

| **Variables** | **Single-task** | | **Dual-task** | |
| --- | --- | --- | --- | --- |
|  | **Older group** | **Young group** | **Older group** | **Young group** |
| SD__R_AP_ ^T^ | 0.047±0.026 | 0.037±0.020 | 0.083±0.059 | 0.059±0.035 |
| SD__R_ ML_ | 0.40±0.10 | 0.37±0.16 | 0.47±0.17 | 0.44±0.24 |
| VEL__R_AP_ (cm/s) ^G & T^ | 0.14±0.041 | 0.11±0.022 | 0.23±0.11 | 0.15±0.025 |
| VEL__R_ ML_ (cm/s) ^G & T^ | 0.83±0.21 | 0.53±0.12 | 1.27±0.53 | 0.88±0.25 |
| SA__R_ (cm^2^) | 0.15±0.060 | 0.13±0.13 | 0.62±0.68 | 0.34±0.49 |
| SD__L_AP_ ^T^ | 0.048±0.015 | 0.035±0.011 | 0.082±0.040 | 0.064±0.031 |
| SD__L_ ML_ | 0.39±0.087 | 0.34±0.12 | 0.48±0.16 | 0.42±0.28 |
| VEL__L_AP_ (cm/s) ^G, T & I^ | 0.15±0.027 | 0.11±0.032 | 0.26±0.12 | 0.15±0.043 |
| VEL__L_ ML_ (cm/s) ^G & T^ | 0.75±0.20 | 0.49±0.13 | 1.33±0.66 | 0.79±0.32 |
| SA__L_ (cm^2^) ^T^ | 0.17±0.10 | 0.10±0.054 | 0.52±0.46 | 0.38±0.47 |
| SD_AP_ | 0.39±0.089 | 0.35±0.12 | 0.45±0.089 | 0.35±0.12 |
| SD _ML_ ^G & T^ | 0.12±0.029 | 0.081±0.026 | 0.21±0.12 | 0.14±0.062 |
| VEL_AP_ (cm/s) ^G & T^ | 0.79±0.18 | 0.51±0.083 | 1.25±0.48 | 0.83±0.26 |
| VEL_ML_ (cm/s) ^G & T^ | 0.40±0.083 | 0.31±0.077 | 0.66±0.26 | 0.45±0.11 |
| SA (cm^2^) ^T^ | 0.80±0.31 | 0.49±0.20 | 2.05±1.80 | 1.22±1.28 |
| ROM__R_AP_ (˚) ^T^ | 0.68±0.22 | 0.69±0.39 | 1.21±1.01 | 1.13±0.78 |
| ROM__R_ ML_ (˚) ^T^ | 0.35±0.13 | 0.32±0.12 | 0.69±0.38 | 0.48±0.27 |
| ROM__L_AP_ (˚) ^T^ | 0.60±0.17 | 0.66±0.36 | 0.95±0.74 | 1.07±0.81 |
| ROM__L_ ML_ (˚) ^T^ | 0.36±0.14 | 0.32±0.16 | 0.59±0.29 | 0.48±0.28 |
| Stiff__R_AP_ (Nm/kg*deg) ^T^ | 1.82±1.02 | 1.60±0.82 | 1.23±0.92 | 1.20±0.71 |
| Stiff__R_ ML_ (Nm/kg*deg) ^T^ | 3.68±1.49 | 5.69±2.64 | 2.47±1.27 | 3.55±1.88 |
| Stiff__L_AP_ (Nm/kg*deg) ^T^ | 1.27±0.58 | 1.39±0.48 | 1.02±0.52 | 1.15±0.62 |
| Stiff__L_ ML_ (Nm/kg*deg) ^T^ | 3.69±2.21 | 6.21±2.39 | 2.92±2.02 | 3.98±1.47 |

R means right side; L means left side; SD means standard deviation of center of pressure (CoP); VEL means average of velocity of CoP; SA means sway aera; ROM means ankle joint range of motion; Stiff means ankle joint stiffness; AP means sagittal plane; and ML means frontal plane. ^G^ Indicates a significant group difference in the follow-up ANOVA analysis. ^T^ Indicated a significant task difference in the follow-up ANOVA analysis. ^I^ Indicated a significant interaction difference in the follow-up ANOVA analysis.

**References**

Chua MC, Hyngstrom AS, Ng AV, Schmit BD (2014) Movement strategies for maintaining standing balance during arm tracking in people with multiple sclerosis. J Neurophysiol 112:1656-1666. <https://doi.org/10.1152/jn.00598.2013>

Duarte M, Zatsiorsky VM (2002) Effects of body lean and visual information on the equilibrium maintenance during stance. Exp Brain Res 146:60-69. <https://doi.org/10.1007/s00221-002-1154-1>

Joshi V, Rouse EJ, Claflin ES, Krishnan C (2021) How does ankle mechanical stiffness change as a function of muscle activation in standing and during the late stance of walking? IEEE Trans Biomed Eng 69:1186-1193. <http://DOI:10.1109/TBME.2021.3117516>

Menegoni F, Galli M, Tacchini E, Vismara L, Cavigioli M, Capodaglio P (2009) Gender‐specific effect of obesity on balance. Obesity 17:1951-1956. <https://doi.org/10.1038/oby.2009.82>

Oyama S, Sosa A, Campbell R, Ortega C, Douphrate DI (2017) Evaluation of upper body kinematics and muscle activity during milking attachment task. Int J Ind Ergon 61:101-106. <https://doi.org/10.1016/j.ergon.2017.05.012>

Winter DA, Prince F, Frank JS, Powell C, Zabjek KF (1996) Unified theory regarding A/P and M/L balance in quiet stance. J Neurophysiol 75:2334-2343. <https://doi.org/10.1152/jn.1996.75.6.2334>
